# Temporal downregulation of the polyubiquitin gene *Ubb* affects neuronal differentiation, but not maturation, in cells cultured *in vitro*

**DOI:** 10.1038/s41598-018-21032-6

**Published:** 2018-02-08

**Authors:** Byung-Kwon Jung, Chul-Woo Park, Kwon-Yul Ryu

**Affiliations:** Department of Life Science, University of Seoul, Seoul, 02504 Republic of Korea

## Abstract

Reduced levels of cellular ubiquitin (Ub) pools due to disruption of the polyubiquitin gene *Ubb* lead to dysregulation of neural stem cell (NSC) differentiation and impaired neuronal maturation in cells isolated from *Ubb*^*−/−*^ mouse embryonic brains. However, it is currently unknown whether Ub is required for the specific stage of neuronal development or whether it plays a pleiotropic role throughout the process. To answer this question, we aimed to downregulate *Ubb* expression temporally during neuronal development, which could not be achieved in *Ubb*^*−/−*^ cells. Therefore, we exploited lentivirus-mediated knockdown (KD) of *Ubb* at different stages of neuronal development, and investigated their phenotypes. Here, we report the outcome of *Ubb* KD on two independent culture days *in vitro* (DIV): DIV1 and DIV7. We observed that NSCs did not differentiate properly via *Ubb* KD on DIV1, but the maturation of already differentiated neurons was intact via *Ubb* KD on DIV7. Intriguingly, *Ubb* KD activated Notch signaling when it had been suppressed, but exerted no effect when it had already been activated. Therefore, our study suggests that Ub plays a pivotal role in NSC differentiation to suppress Notch signaling, but not in the subsequent maturation stages of neurons that had already been differentiated.

## Introduction

Ubiquitin (Ub) is one of the most abundant eukaryotic proteins involved in post-translational modifications^1–3^. Ubiquitylation of target substrates occurs via the actions of three enzymes: E1 Ub-activating enzymes, E2 Ub-conjugation enzymes, and E3 Ub ligases^4,5^. The fate of the substrates is determined by the type of ubiquitylation, i.e., monoubiquitylation or polyubiquitylation with a specific Ub chain linkages^6,7^. The best-known and most important outcome is the polyubiquitylation of substrates with Lys 48 (K48) linkages and their targeting to the 26S proteasome for degradation^8,9^. A timely degradation of substrates is also important for the differentiation of neural stem cells (NSCs), onset of neurogenesis, neuronal development, and neuronal function^10,11^. Indeed, reduced levels of cellular Ub via the disruption of the polyubiquitin gene *Ubb* compromised the degradation of substrates and resulted in the dysregulation of NSC differentiation with inhibition of neurogenesis and impaired neuronal maturation^12–14^.

NSC differentiation is mainly regulated by Notch signaling^15,16^. Typically, Notch signaling is suppressed to promote neurogenesis during embryonic stages, while it is activated to promote gliogenesis and neuronal maturation during postnatal stages^17–20^. Notch signaling is initiated by the interaction between the Notch receptor and its ligand Delta (DLL1) in the neighboring cells, followed by the cleavage and release of Notch intracellular domain (NICD) via γ-secretase. NICD then translocates into the nucleus to form a transactivator complex and activates the transcription of target genes^21^. Notch target genes such as the Hairy/enhancer of split (Hes) and Hairy/enhancer-of-split related with YRPW motif protein (Hey) families are basic helix-loop-helix (bHLH)-type transcription factors that suppress the expression of neurogenic genes^22^. Therefore, in our *in vitro* culture system using cells isolated from embryonic brains on 14.5 days post-coitum (dpc), Notch signaling should be suppressed during the early stage of culture *in vitro*, but it was expected to become activated as the culture progressed. However, in *Ubb*^*−/−*^ cells, increased steady-state levels or delayed degradation of NICD resulted in the activation of Notch signaling even before the start of culture *in vitro*, which was sufficient to reverse the neurogenic and gliogenic potentials of NSCs^12^.

In our previous studies, we used *Ubb*^*−/−*^ cells, in which cellular Ub levels are reduced throughout the neuronal development; therefore, the requirement of Ub for a specific process could not be investigated^12,13^. Although we were able to establish a link between cellular Ub levels and neuronal development, most importantly, we could not answer whether the impaired neuronal maturation was caused by the reduced Ub levels during maturation process or by the defects residing in the neurons generated under Ub deficiency. Therefore, we surmised that temporal reduction of Ub levels via downregulation of *Ubb* during culture *in vitro* may be necessary to resolve these issues.

Herein, we introduced a lentivirus-mediated temporal knockdown (KD) of *Ubb* in cells cultured *in vitro* to overcome the limitation of previous studies that used *Ubb*^*−/−*^ cells. *Ubb* KD on culture days *in vitr*o 1 (DIV1) recapitulated the neuronal phenotypes of *Ubb*^*−/−*^ cells. However, *Ubb* KD on DIV7 exerted no effect on the already differentiated neurons. Therefore, our data suggest that maintenance of Ub levels are important to suppress Notch signaling during early stage of neurogenesis or generation of neurons from NSCs, but not for neuronal maturation, if the neurons were generated under sufficient supply of cellular Ub levels.

## Results

### Dysregulation of NSC differentiation in cells cultured *in vitro* via *Ubb* KD on DIV1

**Figure 1:**
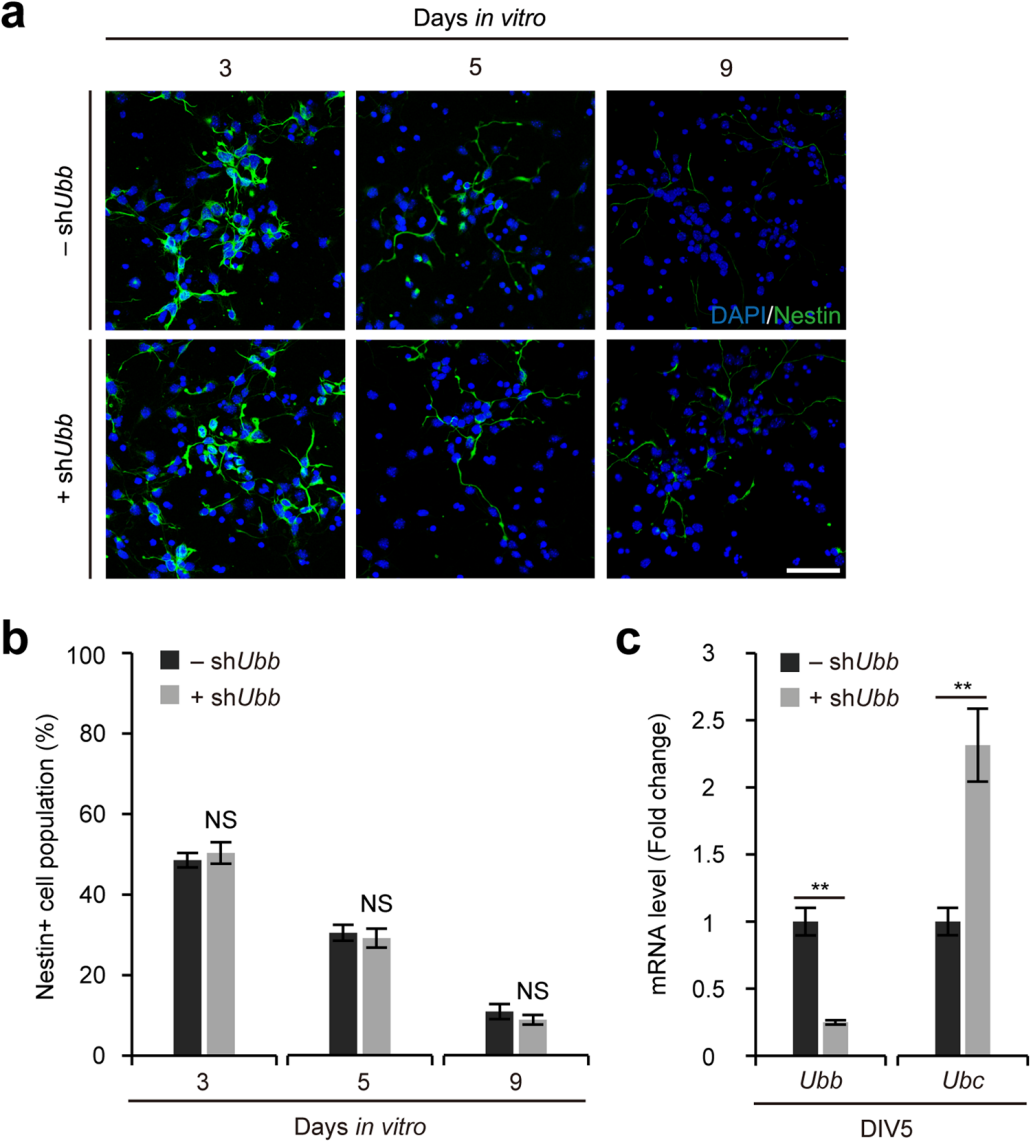
No effect on the exit from NSC’s multipotency during culture *in vitro* via *Ubb* KD on DIV1. (**a**) Cells isolated from embryonic brains on 14.5 dpc (*n* = 3) were infected with lentivirus harboring scrambled RNA (−sh*Ubb*) or *Ubb* shRNA (+sh*Ubb*) on DIV1 and cultured for another 2 to 8 days (DIV3 to DIV9). Cells were fixed and subjected to immunofluorescence analysis using NSC marker nestin. DNA was visualized with DAPI. (**b**) To determine the percentage of nestin+ cell populations, the number of nestin+ cells was divided by the number of DAPI+ cells in three randomly selected fields for each sample (*n* = 3). For each sample, more than 50 DAPI+ cells were counted (>200 cells total). (**c**) Cells were infected with lentivirus harboring scrambled RNA (−sh*Ubb*) or *Ubb* shRNA (+sh*Ubb*) on DIV1 and cultured for another 4 days (DIV5). *Ubb* and *Ubc* mRNA levels in control (−sh*Ubb*) and *Ubb* KD (+sh*Ubb*) cells were determined by qRT-PCR (*n* = 3 each), normalized against *Gapdh* levels, and expressed as a fold change relative to control levels. Representative images of cells from three different embryonic brains are shown (**a**), and data are expressed as the means ± SEM from the indicated number of samples (**b**,**c**). ^**^P < 0.01 between two groups as indicated by horizontal bars. NS, not significant. Scale bar, 50 μm.

When cells were isolated from embryonic brains on 14.5 dpc, two-thirds of them were NSCs^12^. Upon culture in the neuronal growth medium, these cells differentiated into neurons^13^. In fact, immunofluorescence analysis using the NSC marker nestin showed the gradual decrease of the number of NSCs as culture progressed (Fig. 1a,b). On DIV1, cells were infected with the lentivirus harboring sh*Ubb* to investigate how reduced levels of cellular Ub pools via *Ubb* KD affect the differentiation of NSCs into neurons. After 4 days of infection (on DIV5), we confirmed the efficient knockdown of *Ubb* (Fig. 1c). *Ubb* KD did not affect NSC differentiation capacity *per se*, as *Ubb* KD cells also showed the decreased number of NSCs comparable to those of control cells as culture progressed (see Fig. 1b). As previously reported in *Ubb*^*−/−*^ cells^13^, upregulation of the polyubiquitin gene *Ubc* was also observed in *Ubb* KD cells (Fig. 1c). Given that the coding potential of *Ubb*, i.e., the mRNA level multiplied by the number of coding units, is about five times higher than that of *Ubc* in these cells^13^, it is unlikely that ~2-fold upregulation of *Ubc* can compensate for the reduced expression of *Ubb* to maintain the levels of cellular Ub pools.

**Figure 2:**
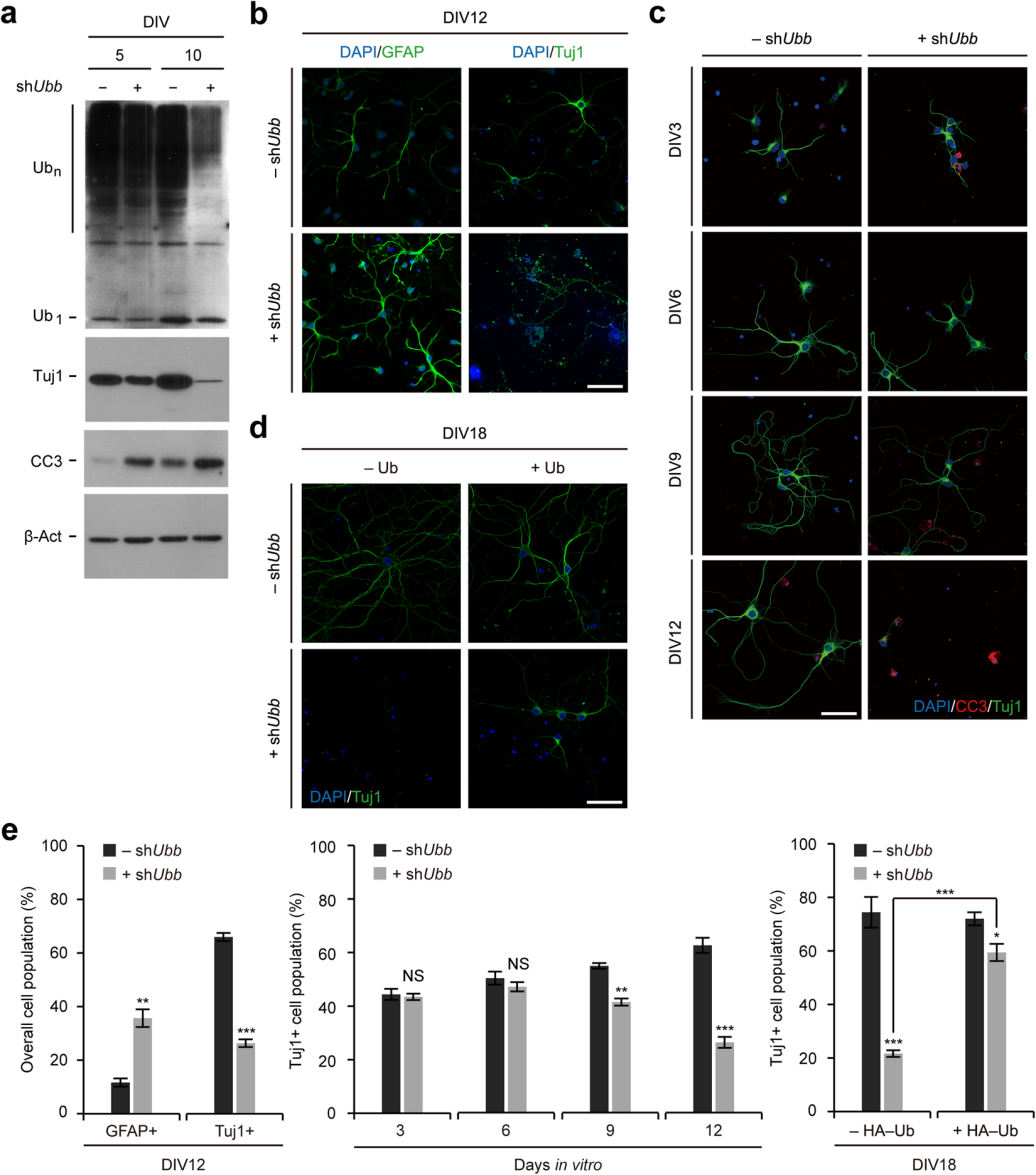
Recapitulation of *Ubb*^*−/−*^ neuronal phenotypes in cells cultured *in vitro* via *Ubb* KD on DIV1. (**a**) Immunoblot detection of Ub conjugates (Ub_n_), free Ub (Ub_1_), neuronal marker Tuj1, and apoptotic marker CC3 in cells infected with lentivirus harboring scrambled RNA (−sh*Ubb*) or *Ubb* shRNA (+sh*Ubb*) on DIV1 and cultured for another 4 or 9 days (DIV5 or DIV10). β-Actin (β-Act) was used as a loading control. (**b**,**c**) Cells were infected with lentivirus harboring scrambled RNA (−sh*Ubb*) or *Ubb* shRNA (+sh*Ubb*) on DIV1 and cultured for another 2 to 11 days (DIV3 to DIV12). Cells were fixed and subjected to immunofluorescence analysis using neuronal marker Tuj1, glial marker GFAP, and apoptotic marker CC3. DNA was visualized with DAPI. (**d**) Cells were infected with lentivirus harboring scrambled RNA (−sh*Ubb*) or *Ubb* shRNA (+sh*Ubb*), and Ub (+Ub) or an empty lentiviral vector (−Ub), on DIV 1 and cultured for another 17 days (DIV18). On DIV18, cells were subjected to immunofluorescence analysis using neuronal marker Tuj1 and DNA was visualized with DAPI. (**e**) The percentage of GFAP+ or Tuj1+ cell populations (*n* = 3) was determined in a similar manner as described in Fig. 1(b). Representative images or immunoblot results of cells from three different embryonic brains are shown (**a**–**d**), and data are expressed as the means ± SEM from the indicated number of samples (**e**). ^**^P < 0.01; ^***^P < 0.001 between two groups as indicated by horizontal bars. NS, not significant. Scale bars, 50 μm.

Accordingly, *Ubb* KD resulted in lower levels of cellular Ub pools, particularly Ub conjugates on DIV10 after prolonged knockdown of *Ubb* (Fig. 2a). Immunoblot analysis using the neuronal marker βIII-tubulin (TUJ1) and the apoptotic marker cleaved caspase-3 (CC3) revealed decreased Tuj1 levels and increased apoptosis in *Ubb* KD cells on DIV5 and DIV10 (Fig. 2a). Immunofluorescence analysis using the neuronal marker Tuj1 and the astrocyte marker glial fibrillary acidic protein (GFAP) showed the defective neuronal development and the increased number of GFAP+ astrocytes in *Ubb* KD cells on DIV12; which was accompanied by the increased number of CC3+ apoptotic cells (Fig. 2b,c,e). It was also obvious that these phenotypes of *Ubb* KD cells aggravated as culture progressed to DIV12 (Fig. 2c,e). However, the simple ectopic expression of Ub improved the neuronal phenotypes of *Ubb* KD cells (Fig. 2d,e). These results suggest that *Ubb* KD at the beginning of cell culture (DIV1) affected NSC differentiation, which promoted gliogenesis and suppressed neurogenesis. In addition, neurons generated under this condition exhibited impaired neuronal maturation and apoptosis. All of these phenotypes are reminiscent of *Ubb*^*−/−*^ cells cultured *in vitro*^12,13^. Therefore, *Ubb* KD on DIV1 is sufficient to recapitulate the neuronal phenotypes of *Ubb*^*−/−*^ cells.

### No effect on neuronal maturation in cells cultured *in vitro* via *Ubb* KD on DIV7

**Figure 3:**
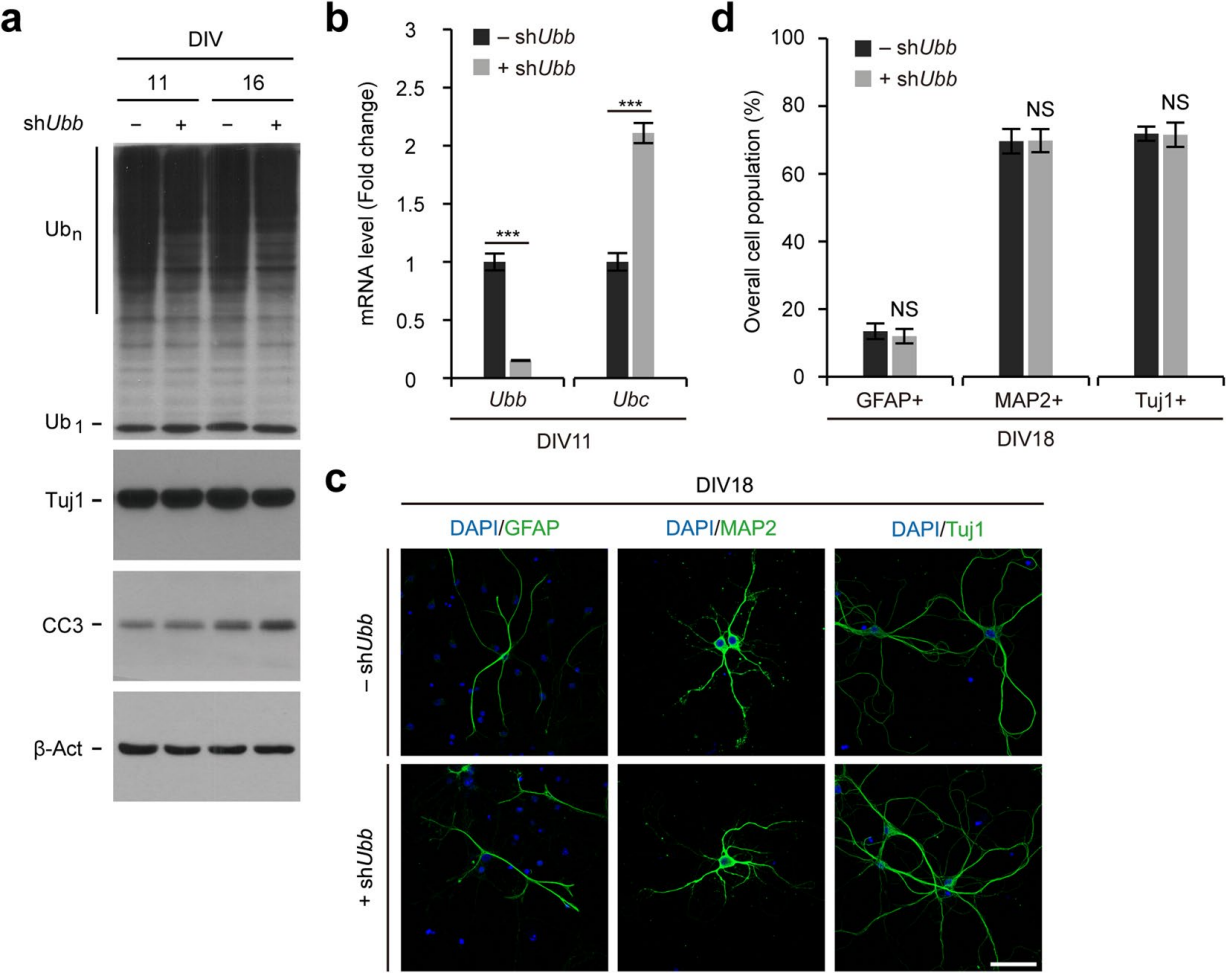
Observation of neuronal maturation in cells cultured *in vitro* via *Ubb* KD on DIV7. (**a**) Immunoblot detection of Ub conjugates (Ub_n_), free Ub (Ub_1_), neuronal marker Tuj1, and apoptotic marker CC3 in cells infected with lentivirus harboring scrambled RNA (−sh*Ubb*) or *Ubb* shRNA (+sh*Ubb*) on DIV7 and cultured for another 4 or 9 days (DIV11 or DIV16). β-Actin (β-Act) was used as a loading control. (**b**) Cells were infected with lentivirus harboring scrambled RNA (−sh*Ubb*) or *Ubb* shRNA (+sh*Ubb*) on DIV7 and cultured for another 4 days (DIV11). *Ubb* and *Ubc* mRNA levels in control (−sh*Ubb*) and *Ubb* KD (+sh*Ubb*) cells were determined by qRT-PCR (*n* = 3 each), normalized against *Gapdh* levels, and expressed as a fold change relative to control levels. (**c**) Cells were infected with lentivirus harboring scrambled RNA (−sh*Ubb*) or *Ubb* shRNA (+sh*Ubb*) on DIV7, cultured for another 11 days (DIV18), and subjected to immunofluorescence analysis using neuronal marker Tuj1, glial marker GFAP, and neuronal maturation marker MAP2. DNA was visualized with DAPI. (**d**) The percentage of GFAP+, MAP2+, or Tuj1+ cell populations (*n* = 3) was determined in a similar manner as described in Fig. 1(b). Representative images or immunoblot results of cells from three different embryonic brains are shown (**a**,**c**), and data are expressed as the means ± SEM from the indicated number of samples (**b**,**d**). ^***^P < 0.001 between two groups as indicated by horizontal bars. NS, not significant. Scale bar, 50 μm.

Neurons generated under Ub deficiency via *Ubb* KD on DIV1 could have already been defective, hampering normal maturation and inducing apoptosis. Therefore, the direct role of Ub in these processes could not be determined. To overcome this, we cultured cells in normal neuronal growth medium for a week to allow normal neuronal differentiation. Subsequently, on DIV7, cells were infected with the lentivirus harboring sh*Ubb* to investigate whether neuronal maturation was indeed affected by reduced levels of cellular Ub pools (Fig. 3a). After 4 days of infection (on DIV11), we confirmed the efficient knockdown of *Ubb* and concomitant ~2-fold upregulation of *Ubc* (Fig. 3b). However, we detected neither reduced Tuj1 levels nor increased apoptosis in *Ubb* KD cells on DIV11 and DIV16, although Ub conjugate levels decreased slightly (see Fig. 3a).

Interestingly, immunofluorescence analysis showed that maturation of normally developed neurons was not influenced by *Ubb* KD on DIV7, as evidenced by the neuronal marker Tuj1 and the neuronal maturation marker microtubule-associated protein 2 (MAP2) (Fig. 3c,d). Furthermore, in contrast to *Ubb* KD on DIV1, *Ubb* KD on DIV7 did not show an increased number of GFAP+ astrocytes (Fig. 3c,d). Therefore, reduced levels of cellular Ub pools do not seem to affect the maturation of already differentiated neurons. Furthermore, these intact neurons did not undergo apoptosis even under Ub-deficient conditions. These results suggest that maintaining Ub above threshold levels is important for the differentiation of NSCs into neurons, but once intact neurons are generated, Ub levels may not be important for their maturation and survival.

### *Ubb* KD at the beginning of the culture period (DIV1) leads to the activation of Notch signaling

**Figure 4:**
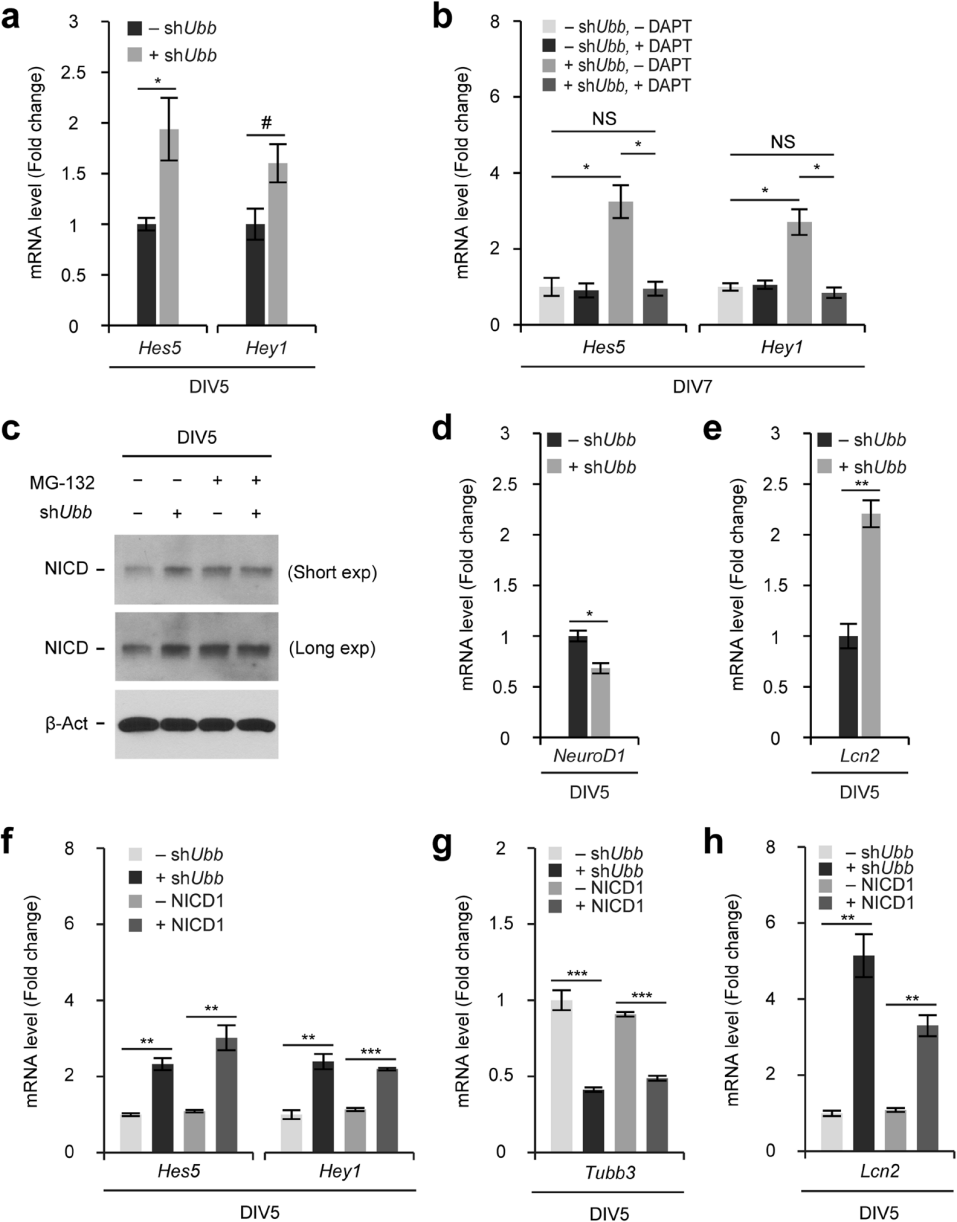
Activation of Notch signaling and its consequences via *Ubb* KD on DIV1. (**a**,**d**,**e**) Cells isolated from embryonic brains on 14.5 dpc (*n* = 3) were infected with lentivirus harboring scrambled RNA (−sh*Ubb*) or *Ubb* shRNA (+sh*Ubb*) on DIV1 and cultured for another 4 days (DIV5). *Hes5*, *Hey1*, *NeuroD1*, and *Lcn2* mRNA levels in control (−sh*Ubb*) and *Ubb* KD (+sh*Ubb*) cells were determined by qRT-PCR (*n* = 3 each), normalized against *Gapdh* levels, and expressed as a fold change relative to control levels. (**b**) Cells were infected with lentivirus harboring scrambled RNA (−sh*Ubb*) or *Ubb* shRNA (+sh*Ubb*) on DIV1, treated with 10 μM DAPT on DIV4 to repress Notch signaling, and cultured for another 3 days (DIV7). *Hes5* and *Hey1* mRNA levels were determined by qRT-PCR (*n* = 3 each), normalized against *Gapdh* levels, and expressed as a fold change relative to control (−sh*Ubb*, −DAPT) levels. (**c**) Immunoblot detection of cleaved NICD in cells infected with lentivirus harboring scrambled RNA (−sh*Ubb*) or *Ubb* shRNA (+sh*Ubb*) on DIV1 and cultured for another 4 days (DIV5). β-Actin (β-Act) was used as a loading control. To prevent potential degradation of NICD, cells were also treated with 10 μM MG-132 for 2 hrs (+MG-132) before harvest on DIV5. (**f**,**g**,**h**) Cells isolated from embryonic brains on 14.5 dpc (*n* = 3) were infected with lentivirus harboring scrambled RNA (−sh*Ubb*), *Ubb* shRNA (+sh*Ubb*), NICD1 (+NICD1), or an empty lentiviral vector (−NICD1) on DIV1 and cultured for another 4 days (DIV5). *Hes5*, *Hey1*, *Tubb3*, and *Lcn2* mRNA levels in control (−sh*Ubb* or −NICD1), *Ubb* KD (+sh*Ubb*), and NICD1-overexpressing (+NICD1) cells were determined by qRT-PCR (*n* = 3 each), normalized against *Gapdh* levels, and expressed as a fold change relative to control levels. Representative immunoblot results of cells from three different embryonic brains are shown (**c**), and data are expressed as the means ± SEM from the indicated number of samples (**a**,**b**,**d**–**h**). ^#^*P* < 0.1; ^*^P < 0.05; ^**^P < 0.01; ^***^P < 0.001 between two groups as indicated by horizontal bars.

It is known that Notch signaling controls the timing of NSC differentiation into neurons and glial cells^16^. Notch signaling is downregulated to promote neurogenesis from NSCs, but once neurons are generated, it is upregulated to promote neuronal maturation and to induce gliogenesis. Aberrant activation of Notch signaling could result in the premature gliogenesis and generation of defective neurons, as observed in *Ubb*^*−/−*^ cells^12^. As *Ubb* KD on DIV1 recapitulated the phenotypes of *Ubb*^*−/−*^ cells, we were curious whether *Ubb* KD on DIV1 also affected Notch signaling. As expected, *Ubb* KD on DIV1 resulted in the increased expression levels of Notch target genes *Hes5* and *Hey1* on DIV5 (Fig. 4a,f). These genes encode inhibitory bHLH transcription factors that inhibit the function of proneuronal bHLH transcription factors and consequently inhibit neuronal differentiation^22,23^.

As in *Ubb*^*−/−*^ cells^12^, upon treatment with DAPT, which is a γ-secretase inhibitor that blocks cleavage of NICD and Notch signaling, we found that the expression levels of Notch target genes in *Ubb* KD cells decreased to those of control cells (Fig. 4b). Therefore, upregulation of Notch target genes in *Ubb* KD cells could be due to increased levels of cleaved NICD. By immunoblot analysis, we confirmed that NICD levels were higher in *Ubb* KD cells than those in control cells (Fig. 4c). Upon inhibition of the proteasome using MG-132, NICD levels increased in control cells, but not in *Ubb* KD cells (Fig. 4c). These results suggest that the increased levels of NICD observed in *Ubb* KD cells are due to the reduced degradation of NICD via the proteasome, as observed in *Ubb*^*−/−*^ cells^12^.

We also measured the expression levels of genes related to neuronal differentiation, *NeuroD1* and *Tubb3* (*Tuj1*), in control and *Ubb* KD cells on DIV5 (Fig. 4d,g). In accordance with the immunoblot or immunofluorescence results, the expression levels of these neurogenic genes significantly decreased in *Ubb* KD cells (Fig. 4d,g). Furthermore, *Lcn2* mRNA levels increased significantly in *Ubb* KD cells (Fig. 4e), suggesting that *Ubb* KD astrocytes were activated to reactive astrocytes, which are known to secrete lipocalin 2 (Lcn2) and may cause the degeneration of defective neurons^24^. Therefore, the neuronal phenotypes observed in *Ubb* KD cells on DIV1 are highly likely due to the activation of Notch signaling, which is supposed to be suppressed to promote neuronal differentiation from NSCs during the early stages of culture *in vitro*.

### Early activation of Notch signaling is sufficient to alter the fate of NSCs

Given that many cellular pathways are involved in the differentiation of NSCs into neurons, *Ubb* KD on DIV1 could have affected pathways other than Notch signaling. If Notch signaling is the major pathway to regulate NSC differentiation, simple upregulation of Notch target genes without diminishing the Ub pool might mimic the outcome of *Ubb* KD on DIV1. To confirm this, we overexpressed Notch intracellular domain 1 (NICD1), which functions as a transcription factor for the expression of Notch target genes, on DIV1 via lentivirus-mediated delivery. NICD1 overexpression or *Ubb* KD on DIV1 increased the expression levels of Notch target genes *Hes5* and *Hey1* to a similar extent (Fig. 4f). Intriguingly, NICD1 overexpression resulted in decreased *Tubb3* (*Tuj1*) mRNA levels and increased *Lcn2* mRNA levels, as observed in *Ubb* KD cells (Fig. 4g,h). Combined with the previous report that the steady-state levels of NICD are increased in *Ubb*^*−/−*^ cells due to delayed degradation^12^, our current data suggest that the activation of Notch signaling via *Ubb* KD on DIV1 is also mediated by the increased NICD levels.

### No further activation of Notch signaling via *Ubb* KD in the middle of the culture period (DIV7)

**Figure 5:**
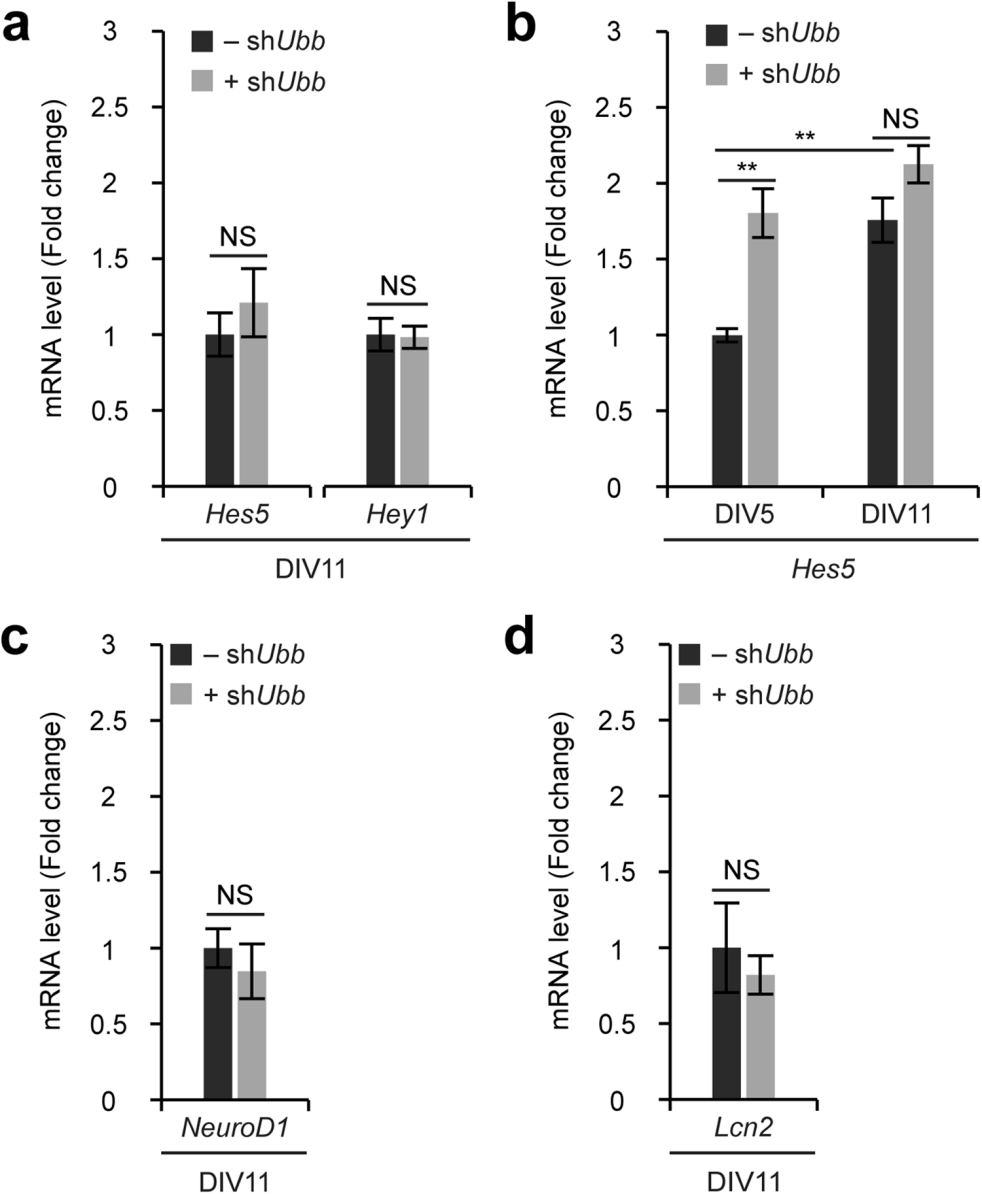
No effect on already activated Notch signaling via *Ubb* KD on DIV7. (**a**,**c**,**d**) Cells isolated from embryonic brains on 14.5 dpc (*n* = 3) were infected with lentivirus harboring scrambled RNA (−sh*Ubb*) or *Ubb* shRNA (+sh*Ubb*) on DIV7 and cultured for another 4 days (DIV11). *Hes5*, *Hey1*, *NeuroD1*, and *Lcn2* mRNA levels in control (−sh*Ubb*) and *Ubb* KD (+sh*Ubb*) cells were determined by qRT-PCR (*n* = 3 each), normalized against *Gapdh* levels, and expressed as a fold change relative to control levels. (**b**) Cells isolated from embryonic brains on 14.5 dpc (*n* = 3) were infected with lentivirus harboring scrambled RNA (−sh*Ubb*) or *Ubb* shRNA (+sh*Ubb*) on DIV1 or DIV7 and cultured for another 4 days (DIV5 or DIV11). *Hes5* mRNA levels in control (−sh*Ubb*) and *Ubb* KD (+sh*Ubb*) cells on DIV5 and DIV11 were determined by qRT-PCR (*n* = 3 each), normalized against *Gapdh* levels, and expressed as a fold change relative to control levels on DIV5. All data are expressed as the means ± SEM from the indicated number of samples. ^**^P < 0.01 between two groups as indicated by horizontal bars. NS, not significant.

As *Ubb* KD on DIV7 did not affect the maturation of already differentiated neurons, we were curious to explore whether it altered Notch target gene expression. To our surprise, *Ubb* KD on DIV7 led to no change in the expression levels of the Notch target genes *Hes5* and *Hey1* on DIV11 (Fig. 5a). Furthermore, the expression levels of *Hes5* were significantly higher on DIV11 than on DIV5. This observation may reflect the fact that Notch signaling becomes upregulated as culture progressed *in vitro* to facilitate neuronal maturation (Fig. 5b). Interestingly, *Ubb* KD on DIV7 did not increase *Hes5* mRNA levels further on DIV11, although *Ubb* KD on DIV1 increased significantly its mRNA levels on DIV5. Furthermore, *Ubb* KD on DIV7 did not also affect the expression levels of *NeuroD1* and *Lcn2* (Fig. 5c,d), which supports the immunoblot and immunofluorescence results obtained using neuronal, glial, and apoptotic markers (see Fig. 3a,c,d).

Based on our results, we conclude that Ub is required for the differentiation of NSCs, but not for the maturation of already differentiated neurons. It seems that the suppression of Notch signaling during the early stages of neurogenesis is facilitated by Ub-mediated NICD degradation. Therefore, reduced levels of cellular Ub antagonize this effect and activate Notch signaling. However, when Notch signaling is activated during the late stages of neurogenesis, NICD is not degraded, but stabilized and translocated into the nucleus to exert its function as a transcription factor; thus, it can no longer be regulated by the levels of cellular Ub pools.

## Discussion

NSCs are differentiated into neurons and astrocytes (or glial cells), in a process where neurogenesis precedes gliogenesis^25,26^. Differentiated neurons undergo neuronal maturation including neuritogenesis and synaptogenesis^10^. We have previously demonstrated that reduced levels of cellular ubiquitin (Ub) in cells isolated from mouse embryonic brains on 14.5 dpc lead to delayed degradation or increased steady-state levels of NICD, resulting in the increased expression of Notch target genes^12^. Activation of Notch signaling during the early stage of neuronal development resulted in (1) dysregulation of NSC differentiation with premature gliogenesis and inhibition of neurogenesis, (2) impaired neuronal maturation, and (3) apoptosis. However, it remained unknown whether reduced levels of Ub have direct influence on each process or whether all phenotypes are the simple outcome of defective neurons generated from dysregulated NSC differentiation. In this study, we attempted to answer this question by infecting cells with the lentivirus harboring sh*Ubb* at the beginning or in the middle of the culture period.

Upon isolation of cells from embryonic brains on 14.5 dpc, two-thirds of the population were NSCs^12^. Based on our experience using the neuronal marker Tuj1 and the neuronal maturation marker MAP2 during culture *in vitro*, these cells actively differentiated into neurons until DIV8. Thereafter, differentiation slowed down, but maturation continued until the end of the culture period (Jung *et al*., unpublished observation). Therefore, we decided to knockdown *Ubb* at two different time points during the culture *in vitro*: DIV1 and DIV7, to determine the direct effect of reduced Ub levels on NSC differentiation into neurons and on neuronal maturation, respectively.

Via *Ubb* KD on DIV1, we were able to determine whether Ub is required for the differentiation of NSCs into neurons. Alternatively, via *Ubb* KD on DIV7, we were able to determine whether Ub is required for the maturation of already differentiated neurons. *Ubb* KD on DIV1 induced the activation of Notch signaling, resulting in decreased expression of neurogenic genes and increased apoptosis. In contrast, *Ubb* KD on DIV7 induced no further activation of Notch signaling, which had already been activated, and no dramatic effects on already differentiated neurons were observed. Furthermore, the overexpression of Ub ameliorated the neuronal phenotypes via *Ubb* KD on DIV1, and the overexpression of NICD1 recapitulated the phenotypes observed via *Ubb* KD on DIV1, such as downregulation of neurogenic genes.

Defective neurons generated via *Ubb* KD on DIV1 or in *Ubb*^*−/−*^ cells are highly susceptible to apoptosis. Defective neurons have been shown to activate astrocytes, and these reactive astrocytes secrete Lcn2, which may be responsible for the neuronal apoptosis^24^. Increased *lcn2* mRNA levels observed in *Ubb* KD cells (see Fig. 4e) or in *Ubb*^*−/−*^ cells (Park *et al*., unpublished data) suggest the activation of astrocytes. As NICD1 overexpression was sufficient to increase *Lcn2* mRNA levels (see Fig. 4h) without diminishing cellular Ub pools, simple alterations of Notch signaling toward increasing the NSC’s gliogenic potential can render neurons defective and activate astrocytes. Under high gliogenic potential, the number of these reactive astrocytes can also increase, and their neurotoxic characters are further enhanced, while their neuroprotective functions are lost^24,27^. Intriguingly, when intact neurons were already generated, *Ubb* KD on DIV7 did not increase *Lcn2* mRNA levels (see Fig. 5d). These results suggest that reactive astrocytosis or increased *Lcn2* expression are not directly caused by Ub deficiency, but are more likely related to the premature gliogenesis induced by aberrant activation of Notch signaling.

It seems that oscillation of Notch target protein levels (e.g., Hes1) may be important for NSCs to maintain their status before differentiating into neurons^23,28,29^. During oscillation, their levels change every few hours, i.e., increase by upregulation of their expression and decrease by proteasomal degradation. Stops in oscillation by maintaining their levels low and maintaining high levels of neurogenic gene expression can induce differentiation into neurons. However, under Ub deficiency or under forced overexpression of NICD1, Notch target protein levels increase constitutively and may affect their oscillation. Under such circumstances, stops in oscillation with high Notch target protein levels favor differentiation into astrocytes instead of neurons, as neurogenic gene expression is downregulated. On DIV7, many NSCs have already been differentiated into neurons. Although we cannot exclude the possibility that *Ubb* KD on DIV7 can still have an effect on remnant NSCs and alter their fate, this effect may be negligible as the cellular microenvironment is already under high Notch signaling activity to promote neuronal maturation.

In conclusion, our data suggest that the maintenance of high levels of cellular Ub pool is required for the differentiation of NSCs into neurons to suppress the Notch signaling pathway. However, it is likely that levels of cellular Ub pool may not be important for the subsequent maturation stages of neurons that have already been differentiated, since cellular Ub pools do not affect Notch signaling when it is activated.

## Methods

### Isolation and culture of mixed neuronal cells

CD-1 (ICR) mice were kept in plastic cages with *ad libitum* access to food and water. All experimental protocols including breeding, euthanasia, and dissection of embryonic brains, were approved by the University of Seoul Institutional Animal Care and Use Committee (UOS IACUC; approval No. UOS-121025-2). All animal procedures were carried out in accordance with relevant guidelines and regulations approved by the UOS IACUC.

Isolation and culture of mixed neuronal cells were carried out essentially as previously described^12^. Briefly, on 14.5 dpc, embryonic brains were dissected and the cerebellum and meninges were removed in Hank’s Balanced Salt Solution (HBSS). Processed brains were transferred to 0.05% trypsin/EDTA (Cellgro), incubated for 30 min at 37 °C with shaking, and an equal volume of cell culture medium (DMEM supplemented with 10% fetal bovine serum [FBS], 20 mM l-glutamine, and 1% antibiotics/antimycotics [Cellgro]) was added to quench trypsinization. After centrifugation, the brain tissue was triturated in neuronal growth medium (Neurobasal® medium supplemented with B-27® supplement [Invitrogen], 1× GlutaMax, 0.5 mM l-glutamine, and 1% antibiotics/antimycotics [Cellgro]) using a 1,000 μl tip, and strained through a 40 μm nylon mesh. The resulting cells were then cultured in the same medium on a cell culture dish coated with poly-D-lysine (MW 30,000–70,000; Sigma-Aldrich) and laminin (Invitrogen). One-half of the medium was changed every 3 days.

### Generation of lentivirus harboring Ub, NICD1, and shRNA against *Ubb*

The pM1.4-MCMV-HA-Ub lentiviral vector was generated as previously described^13^. The pENTR-NICD1 vector was obtained from Addgene (#46048) and a 2,385-bp *Bam*HI/*Eco*RI fragment containing an open reading frame was inserted into the pM1.4-MCMV vector digested with *Bam*HI/*Eco*RI. The pLKO.1-scramble shRNA and pLKO.1-sh*Ubb* were obtained from Addgene (#1864) and Sigma-Aldrich (TRCN0000098636), respectively. The oligonucleotide targeting sequence for mouse *Ubb* was 5′-CGA GAA TGT GAA GGC CAA GAT-3′. Generation of lentivirus was carried out essentially as previously described^13,30^. Briefly, one day before transfection, 293 T cells were plated on 100 mm dishes at 1.5 × 10^6^ cells/dish and incubated in cell culture medium (DMEM supplemented with 10% FBS, 20 mM l-glutamine, and 1% antibiotics/antimycotics). To produce a lentivirus harboring Ub, NICD1, scramble shRNA, or sh*Ubb*, packaging plasmid psPAX2 (8 μg), envelope plasmid pMD2.G (3 μg), and transfer plasmid pLentiM1.4-MCMV-HA-Ub, pM1.4-MCMV-NICD1, pLKO.1-scramble, or pLKO.1-sh*Ubb* (10 μg), were mixed together in 5 mM HEPES (pH 7.3) buffered water in each dish, and transfected using a standard CaPO_4_ method. After incubation for 16 hrs, medium with precipitates was replaced with a fresh medium (DMEM supplemented with 1% FBS, 20 mM l-glutamine, and 1% antibiotics/antimycotics) and incubated for another 24–48 hrs. Subsequently, the medium was collected, filtered through a 0.45-μm low-protein binding filter (Pall Corporation), mixed with Lenti-X™ concentrator (Clontech), and incubated at 4 °C overnight. After incubation, the mixture was centrifuged and the viral pellet was resuspended in phosphate-buffered saline (PBS), titrated using qPCR Lentivirus Titration Kit (ABM), and stored in aliquots at −80 °C.

### Immunofluorescence analysis

Immunofluorescence analysis was carried out essentially as previously described^12^. Briefly, cells grown on poly-D-lysine-coated coverslips were fixed in 4% paraformaldehyde for 10 min at room temperature (RT), permeabilized with 0.3% Triton X-100/PBS, blocked with 3% BSA/PBS for 1 hr at RT, and incubated with anti-Tuj1 (1:1,000, Millipore), anti-GFAP (1:1,000, Millipore), anti-MAP2 (1:1,000, Millipore), anti-nestin (1:1,000, Abcam), or anti-CC3 (1:500, Millipore) antibody at 4 °C overnight, followed by an incubation with Alexa Fluor 488 or 555-conjugated goat anti-mouse or donkey anti-rabbit IgG (1:1,000, Invitrogen) with 0.1 μg/ml of 4′,6-diamidino-2-phenylindole (DAPI) for 1 hr at RT. Prolong Gold antifade reagent (Invitrogen) was used for mounting onto slides and immunofluorescence images were visualized with a Carl Zeiss AxioImager A2 microscope or Carl Zeiss Axiovert 200 M microscope equipped with a confocal laser scanning module LSM510.

### Immunoblot analysis

Immunoblot analysis was carried out as previously described with slight modifications^12^. Briefly, cell lysates were prepared in immunoprecipitation (IP) buffer (50 mM Tris-HCl [pH 7.5], 200 mM NaCl, 1% NP-40, 1% sodium deoxycholate with 1 mM PMSF, 1 μg/μl aprotinin, and 1 μg/μl leupeptin as protease inhibitors) and incubated on ice for 30 min. Total cell lysates (15 μg) were subjected to SDS-PAGE, followed by immunoblot detection with anti-Tuj1 (1:1,000, Millipore), anti-Ub (1:1,000, Millipore), anti-Notch3 (1:200, Santa Cruz Biotechnology), anti-CC3 (1:1,000, Millipore) or anti-β-Actin antibody (1:2,000, Santa Cruz Biotechnology). Based on the types of primary antibodies, the appropriate HRP-conjugated goat anti-mouse or anti-rabbit IgG (1:10,000, Enzo Life Sciences) or HRP-conjugated donkey anti-goat IgG (1:5,000, Santa Cruz Biotechnology) was used.

### Quantitative real-time RT-PCR

Quantitative real-time RT-PCR (qRT-PCR) was carried out as previously described with slight modifications^12^. Briefly, total RNA was isolated from cultured neurons using TRI reagent (Molecular Research Center) or RNeasy plus kit (Qiagen). Before reverse transcription, DNase I (amplification grade, Invitrogen) was treated for 15 min at RT. Reverse transcription was carried out using 1 μg total RNA per sample in a 25 μl reaction using the GoScript™ Reverse Transcription System (Promega) or SuperiorScript II Reverse Transcriptase (Enzynomics) according to manufacturer’s protocol. For qRT-PCR, we used an SYBR qPCR 2× Mastermix (Enzynomics) and iCycler system with iCycler iQ software version 2.0 (Bio-Rad). The mRNA expression levels of *Ubb*, *Ubc*, *Tubb3*, *NeuroD1*, *Hes5*, *Hey1*, and *Lcn2* were normalized to the levels of *Gapdh*. Primers used for qRT-PCR are as follows: *Ubb*-F (5′-TCT GAG GGG TGG CTA TTA A-3′); *Ubb*-R (5′-TGC TTA CCA TGC AAC AAA AC-3′); *Ubc*-F (5′-GTT ACC ACC AAG AAG GTC-3′); *Ubc*-R (5′-GGG AAT GCA AGA ACT TTA TTC-3′); *Tubb3*-F (5′-GCA TGG ATG AGA TGG AGT TC-3′); *Tubb3*-R (5′-TCC GAT TCC TCG TCA TCA TC-3′); *NeuroD1*-F (5′-TGA CCT TTC CCA TGC TGA AT-3′); *NeuroD1*-R (5′-AGT GCT AAG GCA ACG CAA T-3′); *Hes5*-F (5′-GCA GCA TAG AGC AGC TGA AG-3′); *Hes5*-R (5′-AGG CTT TGC TGT GTT TCA GG-3′); *Hey1*-F (5′-AAA ATG CTG CAC ACT GCA GG-3′); *Hey1*-R (5′-CGA GTC CTT CAA TGA TGC TCA G-3′); *Lcn2*-F (5′-CTG AAT GGG TGG TGA GTG TG-3′); *Lcn2*-R (5′-GCT CTC TGG CAA CAG GAA AG-3′); *Gapdh*-F (5′-GGC ATT GCT CTC AAT GAC AA-3′); and *Gapdh*-R (5′-CTT GCT CAG TGT CCT TGC TG-3′).

### Statistical analysis

Two-tailed unpaired Student’s t-tests were used to compare the data between two groups. Although differences were considered statistically significant at *P* < 0.05 in most cases, significance was also set at *P* < 0.1 in some cases.

### Data availability

The datasets generated during and/or analyzed during the current study are available from the corresponding author on reasonable request.

## Electronic supplementary material

Figure S1
