# Supplementary material for: Temporal downregulation of the polyubiquitin gene Ubb affects neuronal differentiation, but not maturation, in cells cultured in vitro
Source: Sci Rep. 2018 Feb 8;8:2629. doi: 10.1038/s41598-018-21032-6 (PMC5805694; doi:10.1038/s41598-018-21032-6)

Supplementary Information

Temporal downregulation of the polyubiquitin gene *Ubb* affects neuronal differentiation, but not maturation, in cells cultured *in vitro*

Byung-Kwon Jung, Chul-Woo Park & Kwon-Yul Ryu*

Department of Life Science, University of Seoul, Seoul 02504, Republic of Korea

*Correspondence to: Kwon-Yul Ryu, Department of Life Science, University of Seoul, 163 Seoulsiripdae-ro, Dongdaemun-gu, Seoul 02504, Republic of Korea.

E-mail: kyryu@uos.ac.kr.

Figure S1. Presentation of original immunoblots.

The cropped parts of immunoblots were indicated with boxes. Asterisks indicate adhesive tapes used to attach X-ray film on the white paper.

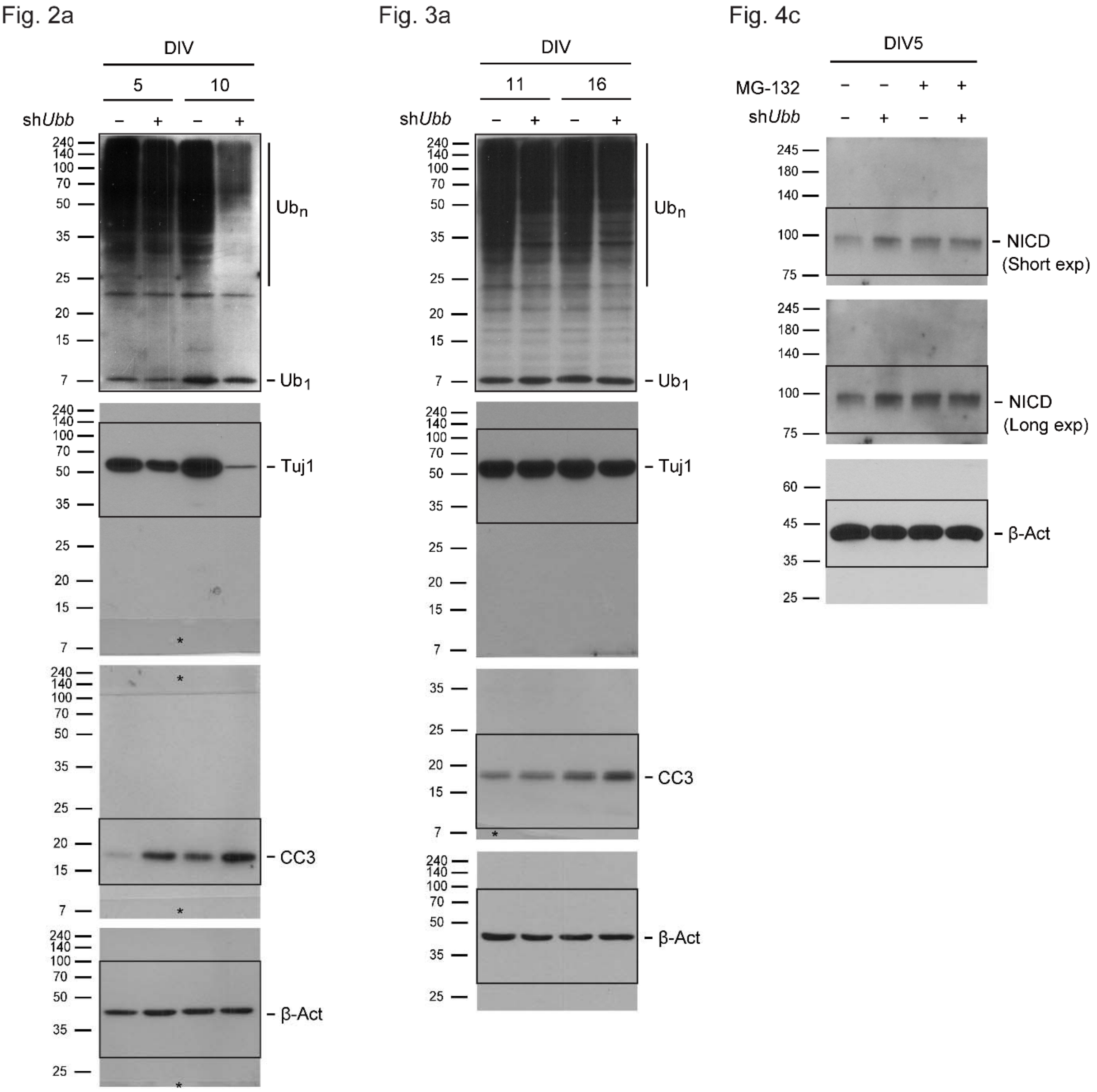

Supplement: Supplementary file 1 — Figure S1 [file 41598_2018_21032_MOESM1_ESM.pdf]
